# Comparative effectiveness of herb-partitioned moxibustion plus lifestyle modification treatment for patients with simple obesity: A study protocol for a randomized controlled trial

**DOI:** 10.1097/MD.0000000000023758

**Published:** 2021-01-22

**Authors:** Li-Hua Wang, Si-Ying Lv, Yi-Ran Liu, Xia Chen, Jia-Jie Wang, Wei Huang, Zhong-Yu Zhou

**Affiliations:** a College of Acupuncture and Orthopedics, Hubei University of Chinese Medicine/Hubei Provincial Collaborative Innovation Center of Preventive Treatment by Acupuncture and Moxibustion; b Department of Acupuncture, Hubei Provincial Hospital of Traditional Chinese Medicine, Wuhan, China.

**Keywords:** a randomized controlled trial, herb-partitioned moxibustion, lifestyle modification treatment, protocol, simple obesity

## Abstract

**Introduction:**

Obesity is a global public health issue, which results in many health complications. Moxibustion may serve as an alternative management for simple obesity, where pharmacological therapy is always difficult to be accepted by the majority of obese patients based on its safety. However, the effects of herb-partitioned moxibustion as obesity intervention have not been confirmed. This study is designed as a single-blinded, 3-dummy randomized controlled trial to evaluate the efficacy and safety of herb-partitioned moxibustion plus lifestyle modification treatment in patients with simple obesity.

**Methods and analysis:**

This study will be a randomized, controlled trial conducted from April, 2019 to April, 2021 that includes 108 participants who have simple obesity and meet the eligibility criteria. The participants will be randomly divided into 3 treatment groups: heat application group, medicated plaster group, or herb-partitioned moxibustion group. Each treatment will last 4 weeks. The primary outcomes will be the clinical effectiveness. The secondary outcome measures include participants’ obesity-related indicators, the IWQOL-Lite scale, and the syndrome score of Traditional Chinese Medicine. Adverse events will be recorded during the intervention period.

**Ethics and dissemination:**

Ethical approval of this study was granted by the Ethics Committee of Hubei Provincial Hospital of Traditional Chinese Medicine on 15 November 2018 (Ethics Reference No: HBZY2018-C24-01). Written informed consents will be provided by all participants before they were enrolled in this study.

**Trial registration number:**

NCT04606680

## 1 Introduction

Obesity, which is resulted from a broken energy homeostasis, is defined as an excessive or abnormal fat accumulation.^[1,2]^ Epidemiological surveys show that obesity has a continuously increasing trend worldwide.^[3,4]^ In 2016, there were over 1.9 billion adults overweight. In the United States, at least 78.6 million people are suffering from obesity.^[5]^ According to WHO, around 2.8 million people die annually due to overweight or obesity. It has been reported that obesity increased the incidence and risks of disorders and diseases such as cardiovascular disease, type 2 diabetes mellitus, neurodegenerative diseases, liver disease, and even cancer, in particular Alzheimer disease.^[6–8]^ In addition, not only the obesity epidemic has a major impact on people's health but many risk factors can also increase obesity prevalence based on the complexity of its pathophysiology, metabolic consequences, and mechanisms.^[9,10]^ Nowadays, overweight and obesity have become an epidemic of the 21st century due to the speed of its development. Research is underway around the world for a better understanding of the pathogenesis and etiology of obesity.

As part of traditional Chinese medicine (TCM), moxibustion interventions have been widely used in clinical practice. Recent studies have shown that moxibustion is effective in adjusting the levels of fat accumulation, blood lipids, and female sex hormones.^[11,12]^ For example, a Chinese research showed that moxibustion stimulates the patient's intestinal micro ecology system by stimulating related acupoints, accelerates the lipid metabolism in the endocrine system and corrects gut microbes disorders, thereby achieving the effect of weight loss.^[13]^ Several studies in animal models have also demonstrated the positive effects of moxibustion in patients with endocrine issues and excess body weight. Specifically, Zhu and colleagues found that moxibustion significantly decreased total plasma cholesterol and slowed the rate of body weight gain in 14-month-old rats when compared with the control group.^[14]^ Overall, these findings suggest that moxibustion has a positive impact on both body weight and blood lipids management. It has become more and more popular with patients and doctors based on its unique advantages of effectiveness, simplicity, and convenience.

To date, there are no studies on the effect of a combined application of herb-partitioned moxibustion plus lifestyle modification treatment in patients with simple obesity. We will conduct a 3-arm parallel clinical trial. In this study, our aim is to investigate the effects of a combined application of herb-partitioned moxibustion plus lifestyle modification treatment in terms of obesity-related indicators, the syndrome score of TCM, and the improvement of the quality of life in patients with simple obesity. We hypothesize that a combination of partitioned moxibustion plus lifestyle modification treatment will produce more beneficial results than the application of these therapies alone in addressing obesity symptoms.

## 2 Methods and analysis

### 2.1 Study design

**Figure 1:**
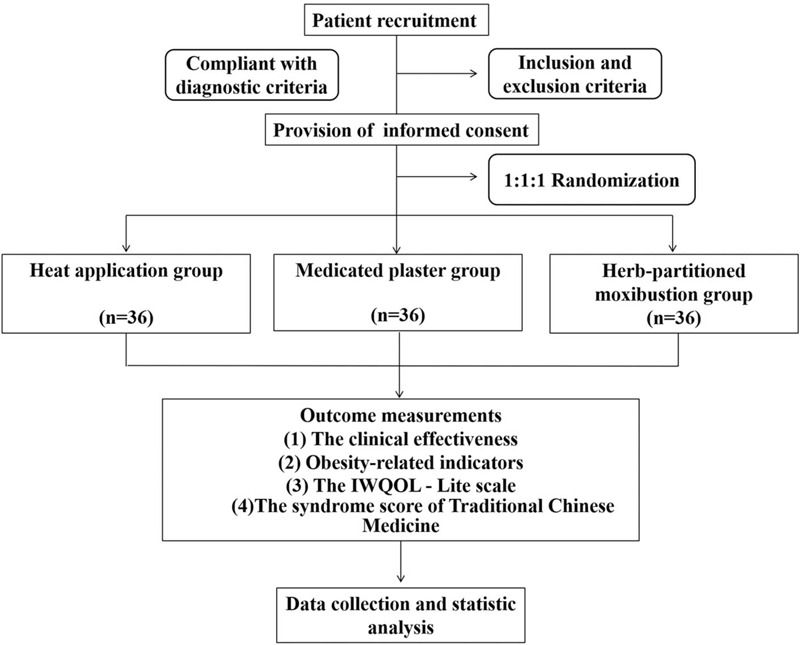
Flow diagram of the study procedure.

This trial is designed as a prospective, outcome-assessor-blinded, parallel-arm, single-center (Hubei Provincial Hospital of TCM, China), randomized controlled clinical trial with a 1:1:1 allocation ratio to evaluate the efficacy of herb-partitioned moxibustion plus lifestyle modification treatment for patients with simple obesity. A total of 108 participants, who meet the inclusion and exclusion criteria, will be randomly allocated to heat application group, medicated plaster group, or herb-partitioned moxibustion group (n = 36 each). The patients will receive these treatments once per day, 3 d/wk for 4 weeks. The flow diagram of the study procedure is showed in Figure 1. Participants’ recruiting is from April 2019.

### 2.2 Recruitment

Trial participants with simple obesity will be recruited at the Hubei Provincial Hospital of TCM in China. The study will be advertised through the internet, posters, and local newspapers in hospitals and communities. The study procedure will be informed to those interested individuals who want to participate in this clinical trial through visits to our hospital or telephone calls. During visits to our hospital, the participants who meet the inclusion and exclusion criteria will be asked to voluntarily sign an informed consent form before participation. Every time the enrolled participant visits, the clinicians of outpatient clinics in our hospital will tell them the next visit's schedule and remind them of the schedule by telephone the day before the visit. In addition, the medical condition of enrolled participants will be continuously monitored by the clinical research coordinator to ensure adherence to the intervention protocols.

### 2.3 Study procedure

This study consists of 2 phases including:

(1) a 4-week screening phase (week 0);
(2) a 4-week treatment phase (week 1–4).

When potential participants show interest in this study, they will be instructed to record their medical history in a screening form. Thereafter, they will be invited to complete an eligibility assessment by trial investigators; next, eligible participants will be enrolled and written informed consent will be provided to them. Participants who meet the inclusion criteria and exclusion criteria will be enrolled, grouped, and given a 4-week interventions. The schedule of enrolments, allocation and assessments is given in Table 1 below.

**Table 1:** The schedule of enrolments, allocation, and assessments.

Phase	Enrolment/allocation	The treatment period			
Timepoint	Wk 0	Wk 1	Wk 2	Wk 3	Wk 4
Informed consent	X				
Eligibility screening	X				
Demographic data	X				
Obesity-related indicators	X	X	X	X	X
The IWQOL-Lite scale	X	X	X	X	X
The syndrome score of TCM	X	X	X	X	X
Record of treatment		X	X	X	X
Drug combination∗	X				
Adverse event	X				
Compliance evaluation	X				
Research completion	X				
Investigator review	X				
Supervisor review	X				

### 2.4 Participants

#### 2.4.1 Diagnostic criteria

(1) Body mass index (BMI): BMI = weight (kg)/height (m^2^), according to the criteria about the weight division of Asian adults in the Redefine and Processing of Obesity in Asia Pacific issued by WHO in February 2000: BMI ≥ 23 for overweight, BMI between 23 and 24.9 for the early stage of obesity, BMI between 23 and 24.9 for I degree of obesity, BMI ≥ 30 for II degree of obesity.
(2) Waist circumference (WC): WC ≥90 cm for male and 80 cm for female.

#### 2.4.2 Inclusion criteria

Participants meeting all of the following criteria will be considered for inclusion:

(1) diagnosed as having simple obesity according to the diagnostic criteria;
(2) aged between 18 and 45 years old;
(3) Waistline: male≥ 95 cm, women≥85 cm;
(4) Being able to fully understand and voluntarily sign informed consent.

#### 2.4.3 Exclusion criteria

Participants will be excluded if they have

(1) Presence of endocrine disorders such as: polycystic ovary syndrome; Cushing syndrome; uncorrected thyroid disease.
(2) Presence of diabetes mellitus, or hypertension, or abnormal liver and kidney functions, or mental diseases.^[15]^
(3) Pregnant or lactating state, women who plan to become pregnant within 4 weeks.
(4) History of bulimia, anorexia, or any other eating disorders.^[16]^
(5) Use of medications in the past 3 months, such as diet drugs, corticosteroids, antidepressants, which may affect weight or appetite.
(6) History of surgical weight loss, postoperative adhesions.
(7) History of participating in a clinical study of weight loss or any other therapies to lose weight in the past 3 months.
(8) Presence of local skin rupture, allergy, and scar constitution.
(9) Unable to cooperate with the research caused by other diseases or reasons.

#### 2.4.4 Dropout criteria

Participants will drop out of the trial under the following conditions:

(1) do not cooperate with the specified treatments or reluctance to continue the trial;
(2) objective factors cause data loss or incompleteness that could influence the record of observation indicators;
(3) serious diseases (such as cardiocerebrovascular, liver and kidney diseases) were found during the treatment;
(4) participants who take drugs which have an impact on body weight or appetite during the treatment;
(5) significant deviation in implementation or large error in protocol; or if participants accept any other way to lose weight during treatment.

### 2.5 Randomization and allocation concealment

The web-based online randomization system to be adopted in this study will be performed by an independent Evidence-based Research Centre at Central South Hospital Affiliated to Wuhan University. Eligible patients will be identified by the attending physicians according to the inclusion and exclusion criteria. Eligible subjects who have written an informed consent will be randomly assigned to 1 of these 3 groups. The randomization list is kept by the research coordinator and biostatistician until the end of the trial, the aim of which is to ensure allocation concealment; all the researchers who evaluate the results above will not be aware of which group the participants have been assigned to. All processes will be recorded and saved as appropriate.

### 2.6 Blinding and informed consent

In this study, the participants will be informed that they will have a 1/3 chance of being allocated to receive 1 of the 3 treatments: heat application, medicated plaster, and herb-partitioned moxibustion. The data statisticians and research staff involved in data collection and data analysis will be blinded to the assignment of participants’ group throughout the entire study. However, the participants and therapists cannot be masked due to the nature of these interventions, but they will be trained not to share any information with other participants, researchers, and the data statistician regarding treatment procedures and responses.

### 2.7 Intervention

#### 2.7.1 Lifestyle modification treatment

The following lifestyle modification are recommended to help to establish healthy habits:

(1) firstly, the basal metabolic value of patients will be calculated based on the formula of BMR according to body weight recommended by FAQ/WHO;
(2) according to the basic metabolic value, we can get the difference between the daily intake of diet calories and exercise consumption calories, that is: the intake of dietary calories-exercise consumption of calories ≤ the basic metabolic value;
(3) according to the difference between the calculated dietary calories and exercise calories, all participants will be prescribed an appropriate diet and exercise programs, which is formulated according to requirements of dietary guidelines for Chinese residents approved and issued by the Standing Council of Chinese Nutrition Society. The proportion of 3 nutrients in total calories comprises of 55% to 65% from carbohydrates, 20% to 30% from oils and 15% from protein, and the energy of the whole day distributed to 3 meals will follow the proportion of 30% for breakfast, 40% for lunch, and 30% for dinner. Meanwhile, all participants will be instructed to record their food and calorie intake daily, and low-to-moderate intensity physical activities such as jogging, walking, and swimming will be instructed to them for at least 5 days per week continually, 30% to 50% of the maximum heart rate reserve value will be required. The maximum heart rate reserve value = the patient's maximum heart rate (220-age)-the patient's static heart rate (the heart rate when the patient wakes up in the morning but has not yet got up).

All patients will be instructed to keep a diet and exercise diary.

The intervention time of lifestyle modification treatment will last for 8 weeks.

#### 2.7.2 Heat application group

The acupoints will be selected as Tianshu, Pishu, shenshu, zhongwan, and zusanli. The location of the acupoints were based on the national GB/T 12346-2006 acupoints standard.^[17]^ The hot sticker will be placed on the patient's acupoints for about 2 hours. This treatment will be applied once every other day, 3 times per week, for 4 consecutive weeks. The treatment will be delayed during the menstrual period.

#### 2.7.3 Medicated plaster group

The acupoints will be selected as Tianshu, Pishu, shenshu, zhongwan, and zusanli. The location of the acupoints were based on the national GB/T 12346-2006 acupoints standard.^[17]^The Chinese traditional medicine (aconite root, dried ginger, evodia rutaecarpa, clove, cinnamon, etc) in moderation will be grinded into powder, and the powder will be added with a certain amount of flour, and finally they will be blended with ginger juice to the appearance of mud. Next, 10 g of the kneaded medicine mud will be taken and applied to the bottom of the ordinary sticker. They will be placed on the patient's acupoints for about 2 hours. The sticker has the same shape as the hot sticker, but it does not have the effect of thermal stimulation and infrared radiation. This treatment will be applied once every other day, 3 times per week, for 4 consecutive weeks. The treatment will be delayed during the menstrual period.

#### 2.7.4 Herb-Partitioned moxibustion group

The acupoints will be selected as Tianshu, Pishu, shenshu, zhongwan, and zusanli. The location of the acupoints were based on the national GB/T 12346-2006 acupoints standard.^[17]^ The Chinese traditional medicine (aconite root, dried ginger, evodia rutaecarpa, clove, cinnamon, etc) in moderation will be grinded into powder, and the powder will be added with a certain amount of flour, and finally they will be blended with ginger juice to the appearance of mud. Next, 10 g of the kneaded medicine mud will be taken and applied to the bottom of the hot sticker. They will be placed on the patient's acupoints for about 2 hours. This treatment will be applied once every other day, 3 times per week, for 4 consecutive weeks. The treatment will be delayed during the menstrual period.

### 2.8 Outcome measures

#### 2.8.1 Primary outcomes.

The clinical effectiveness of 4 weeks of

- Heat application group,
- Medicated plaster group, and
- Herb-partitioned moxibustion group for the improvement of simple obesity will be assessed and determined by the clinical evaluation of nimodipine:

(1) Healing: the clinical symptoms disappear or almost disappear, and the syndrome score is reduced by ≥85%;
(2) Significant effect: the clinical symptoms are obviously improved, and the syndrome score is reduced by <85%, but ≥50%;
(3) Effective: the clinical symptoms have improved, syndrome scores decreased by <50%, but ≥30%;
(4) Invalid: the clinical symptoms were not improved, even worse, and the syndrome score was reduced by <30%.

Integral variation formula (Nimodipine method: [(pre-treatment score-post-treatment score)/pre-treatment score] X100%. This index will be assessed on week 4.

#### 2.8.2 Secondary outcomes

##### 2.8.2.1 Obesity-Related indicators

Weight; BMI (weight/(height); body fat percentage; basic metabolism value; WC; hip circumference(HC); waist-to-hip ratio (WC/HC); Visceral fat area; body fat; and obesity degree will be measured on week 0, 1, 2, 3, and 4.

#### 2.8.3 The IWQOL-Lite scale

The IWQOL-Lite scale will be measured on week 0, 1, 2, 3, and 4. The lower the total score, the lighter the clinical symptoms of the patient.

#### 2.8.4 The syndrome score of TCM

The syndrome score of TCM will be adopted as the criterion of syndrome determination according to the diagnostic and therapeutic evaluation standard of simple obesity revised by the 5th National Obesity Research Conference in 1997. The total score will be then calculated and the unit will be expressed in points. The main clinical symptoms of simple obesity of spleen deficiency and dampness resistance type were divided into 4 levels, which include no (0 point), mild (1 point), medium (2 points) and heavy (3 points). Measurements will be made on week 0, 1, 2, 3, 4.

### 2.9 Safety assessments

When any trial-related adverse events including skin rash, skin allergy, and infection occur during this clinical trial, they will be recorded by the clinicians, and the researchers should make sure that the participant is properly and timely medically treated. Three experts from different fields from Hubei Provincial Hospital of TCM compose an independent Safety Monitoring Board, who has the right to make the final decision to terminate the study. All these experts will monitor the safety and performance of this trial to make sure that the study goes smoothly.

### 2.10 Data management

All researchers including the physicians, data collectors, data entry personnel, outcome assessors, and data statisticians will receive special training about the data management and standard procedure. During the recruitment period, the baseline characteristics of participants will be recorded in case report forms (CRF)s by our data collectors and the data manager will check all these data. When the treatment period completes, the data of all participants will have been recorded on the original CRFs and then 2 data entry personnel will enter these data into Excel spreadsheets independently, following which the data manager will cross-check these data sets to ensure accuracy. A corresponding computer database will be established and the medical records of this clinical trial will be input and stored by researches in a password-protected electronic database, and be kept strictly confidential. A member in the study team who is not involved in these data collection will check the database data and source documents. The hard copies of all the clinical data and CRFs will be stored in a fixed locker.

### 2.11 Sample size calculation

In a previous study, the effective rate of simple obesity in the herb-partitioned moxibustion group was *P*1 = .80. The effective rate of the heat application group was *P*2 = .65, and the effective rate of the medicated plaster group was *P*3 = .6. PASS V.11 software (NCSS, Kaysville, UT) was used to calculate that, based on 80% power to detect a significant difference(α = 0.025, 1 sided), 33 participants will be required for each group. Allowing for a 10% withdrawal rate, we plan to include a total of 108 participants with 36 participants in each group.

### 2.12 Statistical analysis

The data of all participants will be conducted in the final statistical analysis which will be in accordance with the per-protocol principle and the intent-to-treat principle. The data statisticians will be blinded to the group allocation. The SPSS software v19.0 (Statistics 19.0, SPSS, IBM Corp., Chicago, IL) will be used for statistical processing. Measurement data will be presented in the form of mean ± standard deviation, while count data will be expressed as percentage and number. The continuous variables, such as participants’ obesity-related indicators including weight, BMI (weight/height), body fat percentage, basic metabolism value, WC, HC, waist-to-hip ratio (WC/HC), visceral fat area, body fat, and obesity degree will be presented as mean ± SD. Between-group comparisons will be performed by ANOVA or its non-parametric equivalents on changes from baseline to after treatment. Intra-group comparison will be performed by using the Kruskal–Wallis test. All statistical tests will be performed on both sides, and the difference will be considered statistically significant when *P* < .05.

### 2.13 Ethics and dissemination

This study was approved by the Ethics Committee of Hubei Provincial Hospital of TCM on 15 November 2018 (Ethics Reference No:HBZY2018-C24-01). This study was registered at https://register.clinicaltrials.gov/prs/app/action/SelectProtocol?sid=S000ACAK&selectaction=Edit&uid=U00034LN&ts=6&cx=luql35 (ClinicalTrials.govID,NCT04606680). Written informed consents will be provided by all participants before they were enrolled in this study. Their personal information will be collected, and maintained in an independent closet to protect confidentiality before, during, and after the clinical trial.

## 3 Discussion

To ensure the quality of this randomized controlled trial, only participants who meet the inclusion criteria of simple obesity will be recruited. Moreover, participants will be excluded if they have endocrine disorders such as polycystic ovary syndrome, diabetes mellitus, or hypertension, or abnormal liver and kidney functions, or mental diseases. Medications related to these diseases may affect the clinical results by interacting with the planned interventions.

Obesity is a common metabolic and endocrine disease which seriously affects patients’ quality of life, and long-term physical and mental health.^[18]^ The clinical complications associated with this disease are complicated, and the interventions are highly heterogeneous. Different treatment methods also exist for the condition. Although conservative management of obesity which includes lifestyle modification and anti-obesity drugs can reduce body weight to some extent, it still has some limitations owing to considerable drug-related side effects (e.g., headache, dizziness, insomnia, constipation, and gastrointestinal adverse effects), poor compliance and possible weight regain once the treatment stops. The gastrointestinal surgery can greatly reduce excess weight, but only approximately 61.7% population reached the therapeutic goal of weight-loss for that the cost and the risk of postoperative complications are both high, and there are strict indications, which was unsatisfactory.^[18–20]^ Therefore, there is an urgent to seeking help from alternative interventions to treat these obese people who are unsuitable candidates for gastrointestinal surgery and also respond inadequately to conservative treatments.

In the past 50 years, it has been illustrated that obesity is mainly caused by a dysfunctional relationship between Spleen, Liver, Kidney, and the sanjiao (triple energizer), and its pathogenesis is closely related to the blood vessel obstruction by Qi deficiency and phlegm dampness stagnation, which might be caused by various internal and external factors such as stasis of liver Qi, deficiency of spleen, or over intake of greasy flavour. Based on the foundation of this theory, the therapeutic principle of simple obesity is invigorating spleen to remove dampness and relieving Qi stagnancy in Liver to remove blood stasis.^[21,22]^ We designed this clinical trial to evaluate the efficacy and safety of partitioned moxibustion plus lifestyle modification treatment for patients with simple obesity. The participants’ obesity-related indicators, the IWQOL-Lite scale, and the syndrome score of TCM. Adverse events will be recorded and reported.

Moxibustion also has been widely used in China for more than 2500 years. Herb-partitioned moxibustion is a critical component of moxibustion therapy in TCM. It treats diseases by placing a piece of herb formula on the patient's acupuncture points and then igniting a moxibustion tube on the herb formula. While to be different with the traditional partitioned moxibustion, herb-partitioned moxibustion used in this study can not only produce heat stimulation and infrared radiation necessary for moxibustion, but also effectively avoid smoke pollution and open fire danger, and can also play the role of medicine. Therefore, we considered whether this new type of herb-partitioned moxibustion therapy could be more effective in improving related symptoms in patients with simple obesity. There is a lack of relevant clinical evidence in this regard, which we aim to address in this clinical trial. Thus, we will provide supporting evidence for this new type of herb-partitioned moxibustion therapy to treat simple obesity. However, this protocol still has some limitations. Firstly, because of the limited funding and the lack of adequate preliminary studies, this survey has been designed as a single-center pilot study with a small sample size. Secondly, although all outcomes will be measured and recorded by an independent researcher in order to minimize the risk of detection bias, the double-blind procedure cannot be completely adopted for the acupuncture therapist. Nevertheless, we expect that this randomized controlled trial will be able to provide some new evidence regarding this modern new herb-partitioned moxibustion therapy in the treatment of simple obesity.

## Acknowledgments

The authors would like to thank all the patients who participated in this study. The authors are thankful for the support for this study: trial coordinating team, research departments, and statistical analysis staff.

## Author contributions

Li-Hua Wang, Si-Ying Lv, and Yi-Ran Liu designed the study protocol and contributed equally to the manuscript. Li-Hua Wang, Xia Chen, and Jia-Jie Wang reviewed the study protocol and drafted the manuscript. Li-Hua Wang is responsible for the sample size calculation and statistical analysis. Wei Huang, and Zhong-Yu Zhou contributed to the discussion. All authors carefully read and approved the final manuscript.

**Conceptualization:** Zhong-Yu Zhou.

**Data curation:** Wei Huang.

**Formal analysis:** Yi-Ran Liu.

**Investigation:** Jia-Jie Wang.

**Methodology:** Li-Hua Wang, Si-Ying Lv, Xia Chen.
